# Adesão, Conformidade e Persistência no Tratamento do Colesterol Alto

**DOI:** 10.36660/abc.20240652

**Published:** 2024-12-20

**Authors:** Tania L. R. Martinez

**Affiliations:** 1 A Beneficência Portuguesa de São Paulo São Paulo SP Brazil BP - A Beneficência Portuguesa de São Paulo, São Paulo, SP – Brazil

**Keywords:** Adesão, Conformidade, Persistência, Colesterol, Tratamento

A falta de adesão aos medicamentos para redução do colesterol continua sendo uma questão crítica no gerenciamento do risco cardiovascular. Apesar dos avanços no tratamento, muitos pacientes não seguem seus regimes prescritos, levando a maiores riscos de ataque cardíaco, derrame e outros eventos cardiovasculares. Conforme descrito no estudo dos ABC Cardiol,^1^ os desafios de adesão na população brasileira ilustram um problema global. No entanto, existem várias estratégias promissoras para aumentar a adesão, incluindo o envolvimento ativo de farmacêuticos e a distribuição de medicamentos em volumes maiores por meio de sistemas de saúde públicos ou privados.

A adesão à medicação, ou o grau em que os pacientes seguem seus regimes prescritos, é fundamental para atingir resultados de saúde ideais em condições crônicas como colesterol alto. A adesão é a consistência com que os pacientes tomam seus medicamentos ao longo do tempo, enquanto a persistência se refere a quanto tempo eles continuam o tratamento sem interrupção. Medicamentos para baixar o colesterol, particularmente estatinas, são amplamente usados para reduzir o risco cardiovascular, mas a não adesão continua sendo uma barreira para sua eficácia total.^2^

Vários fatores contribuem para a baixa adesão ao tratamento do colesterol alto, incluindo muitos pacientes que lutam para aderir ao tratamento porque o colesterol alto é frequentemente assintomático. Sem efeitos imediatos, os pacientes podem não reconhecer a importância do uso consistente de medicamentos. As estatinas, os medicamentos mais comumente prescritos para baixar o colesterol, podem causar efeitos colaterais, como dores musculares, o que pode desencorajar os pacientes a continuar a terapia. O custo dos medicamentos, particularmente para pacientes de baixa renda, pode levar à descontinuação ou racionamento do tratamento.^3^

Os farmacêuticos desempenham um papel crucial na melhoria da adesão à medicação. Sua posição única na área da saúde permite que eles forneçam atendimento personalizado ao paciente, monitorem o uso da medicação e ofereçam conselhos práticos para abordar preocupações comuns, como efeitos colaterais ou a complexidade dos esquemas de dosagem.^4^

Outra estratégia eficaz para melhorar a adesão é a dispensação de medicamentos em volumes maiores, particularmente por meio de sistemas de saúde públicos ou privados. Quando os pacientes recebem seus medicamentos em um suprimento maior, eles têm menos probabilidade de perder doses devido a desafios logísticos ou à inconveniência de visitas frequentes à farmácia.

Um dos principais motivos para a descontinuação é ficar sem medicação. Quando os medicamentos são dispensados em pequenas quantidades, os pacientes precisam reabastecê-los com frequência, o que aumenta as chances de doses perdidas. Ao fornecer volumes maiores de medicação, como um suprimento para 90 dias em vez de um suprimento para 30 dias, os sistemas de saúde podem ajudar a reduzir essas interrupções e promover a persistência a longo prazo.

Em muitos países, o custo dos medicamentos é uma barreira significativa à adesão. Ao dispensar medicamentos em quantidades maiores, os sistemas de saúde públicos e privados podem oferecer economia de custos aos pacientes, reduzindo a despesa geral do tratamento. A dispensação em massa pode levar a economias de escala, reduzindo o custo por unidade dos medicamentos e tornando-os mais acessíveis para os pacientes, especialmente aqueles com recursos financeiros limitados.

Receber medicamentos em volumes maiores reduz a carga sobre os pacientes de visitar farmácias com frequência, particularmente para aqueles em áreas rurais ou carentes, onde o acesso a instalações de saúde pode ser limitado. Essa conveniência aumentada pode ter um impacto substancial na adesão, pois os pacientes têm menos probabilidade de esquecer ou atrasar as recargas.

Quando os pacientes recebem um suprimento maior de medicamentos, isso reforça a ideia de que o tratamento é um compromisso de longo prazo. Isso pode mudar sua mentalidade de ver o medicamento como uma solução de curto prazo para entender que o uso consistente e de longo prazo é crítico para controlar seus níveis de colesterol e prevenir complicações cardiovasculares.

No sistema de saúde brasileiro, esforços recentes para aumentar o fornecimento de medicamentos aos pacientes por meio de programas públicos têm produzido resultados promissores. O estudo dos ABC Cardiol destaca como essas iniciativas estão ajudando a melhorar as taxas de adesão entre a população.^1^ Ao tornar os medicamentos mais acessíveis e baratos, o Brasil fez avanços significativos no enfrentamento da questão da não adesão, particularmente em áreas rurais onde os pacientes enfrentavam anteriormente desafios para obter recargas regulares.

A decisão do governo de fornecer medicamentos em quantidades maiores por meio de seu sistema de saúde pública também aliviou alguns dos encargos financeiros dos pacientes, melhorando as taxas de persistência. Como resultado, mais pacientes estão permanecendo em seus tratamentos de redução de colesterol por períodos mais longos, reduzindo seus riscos de eventos cardiovasculares.

Concluindo, melhorar a adesão, a conformidade e a persistência no tratamento do colesterol alto requer uma abordagem multifacetada que aborde as várias barreiras que os pacientes enfrentam. O envolvimento de farmacêuticos como participantes ativos no atendimento ao paciente pode aumentar significativamente a adesão por meio de educação, aconselhamento e gerenciamento simplificado de medicamentos. Além disso, dispensar medicamentos em volumes maiores, principalmente por meio de sistemas de saúde públicos ou privados, pode reduzir interrupções no tratamento, diminuir custos e aumentar a conveniência para os pacientes. Ao implementar essas estratégias, os provedores de saúde podem fazer um progresso significativo na melhoria dos resultados de saúde de longo prazo para pacientes com colesterol alto.
