# Supplementary material for: Adesão, Conformidade e Persistência no Tratamento do Colesterol Alto
Source: Arq Bras Cardiol. 2024 Dec 20;121(11):e20240652. [Article in Portuguese] doi: 10.36660/abc.20240652 (PMC11809878; doi:10.36660/abc.20240652)
Supplement: Translation

# Translation: Adherence, Compliance and Persistency in High Cholesterol Treatment

**DOI:** 10.36660/abc.20240652i

**Authors:** Tania L. R. Martinez

**Affiliations:** São Paulo SP Brazil BP - A Beneficência Portuguesa de São Paulo, São Paulo, SP – Brazil

**Keywords:** Adherence, Compliance, Persistence, Cholesterol, Treatment

Lack of adherence to cholesterol-lowering medications remains a critical issue in managing cardiovascular risk. Despite advancements in treatment, many patients fail to follow their prescribed regimens, leading to increased risks of heart attack, stroke, and other cardiovascular events. As described in the study,^1^ adherence challenges in the Brazilian population illustrate a global problem. However, there are several promising strategies for increasing adherence, including the active involvement of pharmacists and the distribution of medications in larger volumes through public or private healthcare systems.

Medication adherence, or the degree to which patients follow their prescribed regimens, is fundamental to achieving optimal health outcomes in chronic conditions like high cholesterol. Compliance is the consistency with which patients take their medication over time, while persistence refers to how long they continue treatment without interruption. Cholesterol-lowering drugs, particularly statins, are widely used to reduce cardiovascular risk, but non-adherence remains a barrier to their full effectiveness.^2^

Several factors contribute to poor adherence to high cholesterol treatment, including many patients who struggle to adhere to treatment because high cholesterol is often asymptomatic. Without immediate effects, patients may not recognize the importance of consistent medication use. Statins, the most commonly prescribed cholesterol-lowering drugs, can cause side effects such as muscle pain, which can discourage patients from continuing therapy. The cost of medications, particularly for low-income patients, can lead to discontinuation or rationing of treatment.^3^

Pharmacists play a crucial role in improving medication adherence. Their unique position in healthcare allows them to provide personalized patient care, monitor medication use, and offer practical advice to address common concerns such as side effects or the complexity of dosing schedules.^4^

Another effective strategy for improving adherence is the dispensation of medications in larger volumes, particularly through public or private healthcare systems. When patients receive their medication in a greater supply, they are less likely to miss doses due to logistical challenges or the inconvenience of frequent pharmacy visits.

One of the primary reasons for discontinuation is running out of medication. When drugs are dispensed in small quantities, patients need to refill them frequently, which increases the chances of missed doses. By providing larger volumes of medication, such as a 90-day supply rather than a 30-day supply, healthcare systems can help reduce these interruptions and promote long-term persistence.

In many countries, the cost of medications is a significant barrier to adherence. By dispensing medications in larger quantities, public and private healthcare systems can offer cost savings to patients, reducing the overall expense of treatment. Bulk dispensing can lead to economies of scale, lowering the per-unit cost of medications and making them more affordable for patients, especially those with limited financial resources.

Receiving medications in larger volumes reduces the burden on patients to frequently visit pharmacies, particularly for those in rural or underserved areas where access to healthcare facilities may be limited. This increased convenience can have a substantial impact on adherence, as patients are less likely to forget or delay refills.

When patients receive a larger supply of medication, it reinforces the idea that treatment is a long-term commitment. This can shift their mindset from seeing the medication as a short-term fix to understanding that consistent, long-term use is critical for managing their cholesterol levels and preventing cardiovascular complications.

In the Brazilian healthcare system, recent efforts to increase the supply of medications to patients through public programs have yielded promising results. The study by ABC Cardiol^1^ report underscores how these initiatives are helping to improve adherence rates among the population. By making medications more accessible and affordable, Brazil has made significant strides in tackling the issue of non-adherence, particularly in rural areas where patients previously faced challenges in obtaining regular refills.

The government's decision to provide medications in larger quantities through its public health system has also alleviated some of the financial burdens on patients, improving persistence rates. As a result, more patients are staying on their cholesterol-lowering treatments for longer periods, reducing their risk of cardiovascular events.

In conclusion, improving adherence, compliance, and persistence in high cholesterol treatment requires a multifaceted approach that addresses the various barriers patients face. The involvement of pharmacists as active participants in patient care can significantly enhance adherence through education, counseling, and simplified medication management. Additionally, dispensing medications in larger volumes, particularly through public or private healthcare systems, can reduce interruptions in treatment, lower costs, and increase convenience for patients. By implementing these strategies, healthcare providers can make meaningful progress in improving long-term health outcomes for patients with high cholesterol.
